# The effect of the WKYMVm peptide on promoting mBMSC secretion of exosomes to induce M2 macrophage polarization through the FPR2 pathway

**DOI:** 10.1186/s13018-021-02321-9

**Published:** 2021-03-03

**Authors:** Wenbo Zhao, Junxian Hu, Qingyi He

**Affiliations:** Department of Orthopedics, Southwest Hospital, Army Medical University (Third Military Medical University), Chongqing, 400038 China

**Keywords:** WKYMVm peptide, mBMSCs, Exosomes, M2 macrophages, Polarization, FPR2 pathway

## Abstract

**Background:**

When multicystic vesicles (precursors of exosomes) are formed in cells, there are two results. One is decomposition by lysosomes, and the other is the generation of exosomes that are transported out through the transmembrane. On the other hand, M2 macrophages promote the formation of local vascularization and provide necessary support for the repair of bone defects. To provide a new idea for the treatment of bone defects, the purpose of our study was to investigate the effect of WKYMVm (Trp-Lys-Tyr-Met-Val-D-Met-NH2) peptide on the secretion of exosomes from murine bone marrow-derived MSCs (mBMSCs) and the effect of exosomes on the polarization of M2 macrophages.

**Methods:**

The WKYMVm peptide was used to activate the formyl peptide receptor 2 (FPR2) pathway in mBMSCs. First, we used Cell Counting Kit-8 (CCK-8) to detect the cytotoxic effect of WKYMVm peptide on mBMSCs. Second, we used western blotting (WB) and quantitative real-time polymerase chain reaction (qRT-PCR) to detect the expression of interferon stimulated gene 15 (ISG15) and transcription factor EB (TFEB) in mBMSCs. Then, we detected lysosomal activity using a lysozyme activity assay kit. Third, we used an exosome extraction kit and western blotting to detect the content of exosomes secreted by mBMSCs. Fourth, we used immunofluorescence and western blotting to count the number of polarized M2 macrophages. Finally, we used an inhibitor to block miRNA-146 in exosomes secreted by mBMSCs and counted the number of polarized M2 macrophages.

**Results:**

We first found that the WKYMVm peptide had no toxic effect on mBMSCs at a concentration of 1 μmol/L. Second, we found that when the FPR2 pathway was activated by the WKYMVm peptide in mBMSCs, ISG15 and TFEB expression was decreased, leading to increased secretion of exosomes. We also found that lysosomal activity was decreased when the FPR2 pathway was activated by the WKYMVm peptide in mBMSCs. Third, we demonstrated that exosomes secreted by mBMSCs promote the polarization of M2 macrophages. Moreover, all these effects can be blocked by the WRWWWW (WRW4, H-Trp-Arg-Trp-Trp-Trp-Trp-OH) peptide, an inhibitor of the FPR2 pathway. Finally, we confirmed the effect of miRNA-146 in exosomes secreted by mBMSCs on promoting the polarization of M2 macrophages.

**Conclusion:**

Our findings demonstrated the potential value of the WKYMVm peptide in promoting the secretion of exosomes by mBMSCs and eventually leading to M2 macrophage polarization. We believe that our study could provide a research basis for the clinical treatment of bone defects.

## Introduction

Many diseases can lead to bone defects or poor bone formation, including fractures, bone tumors, and osteoporosis [1, 2]. At present, studies have demonstrated that M2 macrophages function to promote the T helper 2 cell (Th2) immune response and participate in tissue remodeling, the anti-inflammatory response, fibrosis formation, tumor development, and other pathological processes [3–5]. M2 macrophages can downregulate the immune response by secreting the inhibitory cytokines interleukin-10 (IL-10) or transforming growth factor-β (TGF-β) and promote local vascularization, thus providing a choice for the treatment of bone defects [6, 7]. Many local or systemic factors, such as inflammation, can affect the occurrence and development of vascularization. Related literature shows that in the process of vascularization, M2 macrophage polarization can participate in the immunoregulation of mesenchymal stem cells (MSCs) and functional regulation of osteoblasts [8]. Vascularization is a complex process that requires many cytokines, and M2 macrophages promote angiogenesis [9]. According to relevant research, we propose the following hypotheses. First, ISG15 is an important inflammatory-related ubiquitin-like protein. When ISG15 expression is increased, it can decompose multicystic vesicles by lysosomes [10–12]. To increase the secretion of exosomes, we assume that we can reduce the expression of ISG15 by activating the FPR2 pathway in mBMSCs. Second, when TFEB expression is increased, lysosomal activity is increased [13]. Therefore, we assume that we can activate the FPR2 pathway to decrease TFEB expression, which reduces lysosomal activity and increases exosome secretion. If FPR2 pathway activation promotes the secretion of exosomes by mBMSCs, we propose the final hypothesis. Specifically, when the number of exosomes secreted by mBMSCs is increased, M2 macrophage polarization is promoted.

In recent years, promoting local vascularization has been considered a new method to solve bone defects [14]. As a group of cells with plasticity and pluripotency, macrophages exhibit obvious functional differences under the influence of different microenvironments in vivo and in vitro [15]. According to their different activated states and functions, macrophages can differentiate into M1 macrophages and M2 macrophages [16]. M2 macrophages promote local vascularization by secreting cytokines and regulating the immune response [9].

There are three common isoforms of FPRs: FPR1, FPR2, and FPR3 [17]. FPRs can exist in a variety of cells, such as neutrophils and monocytes/macrophages, which secrete a variety of cytokines and participate in a variety of cell behaviors [18]. Among these isoforms, according to our hypotheses, FPR2 pathway activation promotes the secretion of exosomes by inhibiting ISG15 expression and decreasing TFEB expression.

Based on the above rationale, we assumed that FPR2 pathway activation in mBMSCs reduces the decomposition of intracellular multicystic vesicles (precursors of exosomes) and increases the secretion of exosomes. It has also been noted that there are abundant miRNA-146 in exosomes secreted by MSCs, which can lead to M2 macrophage polarization [19–21]. In this study, we explored the mechanism of promoting exosome secretion and M2 macrophage polarization.

## Materials and methods

### Materials and reagents

The mBMSCs were purchased from Wuhan Fine Biotech. The murine bone marrow-derived macrophage cell line RAW264.7 was purchased from American Type Culture Collection (ATCC, Rockville, MD, USA). According to our team’s previous research results [22], we chose the WKYMVm peptide as the activator of the FPR2 pathway. WKYMVm peptide with purity greater than 95% was synthesized by GL Biochem and dissolved in acetonitrile. However, WRW4 peptide, an inhibitor of the FPR2 pathway, was also synthesized by GL Biochem with a purity greater than 95% and dissolved in acetonitrile [22]. F-12 medium, Dulbecco’s modified Eagle’s medium (DMEM), and 0.25% trypsin were purchased from HyClone. Exosome-free fetal bovine serum (FBS) was purchased from Lonsera. Dimethyl sulfoxide (DMSO) was purchased from Sigma-Aldrich. Penicillin and streptomycin were purchased from Beyotime Biotechnology. Phosphate-buffered saline (PBS) solution, 4% paraformaldehyde, Triton X-100 solution, and fluorescent mounting media were purchased from Solarbio. PCR prime and TRIzol buffer were purchased from Invitrogen. RNA reverse transcription kits and fluorescence quantitative PCR kits were purchased from Takara. Radio immunoprecipitation assay (RIPA) dissociation solution, phenylmethanesulfonyl fluoride (PMSF), WB suit, and sealing solution were purchased from Bioss. All primary antibodies, secondary antibodies, and lysozyme activity assay kits were purchased from Abcam. The miRNA-146 inhibitor was purchased from Gene Pharma. Exosome extraction kits and transfection reagents were purchased from QIAGEN. In addition, CCK-8 was purchased from Beyotime Biotechology.

### Cell culture

mBMSCs were cultured in a T25 culture flask filled with 90% F-12 medium and 10% FBS supplemented with penicillin (100 units/mL) and streptomycin (100 mg/mL) in an incubator at 37.0 °C with 5% CO_2_. RAW 264.7 cells were also cultured in a T25 culture flask containing 90% DMEM and 10% FBS supplemented with penicillin (100 units/mL) and streptomycin (100 mg/mL) in an incubator at 37.8 °C with 5% CO_2_. After 24 h, non-adherent cells were removed. After reaching confluence, adherent cells continued to be cultured. The whole incubation process remained unchanged in 37.0 °C with 5% CO_2_ for mBMSCs and 37.8 °C with 5% CO_2_ for RAW 264.7 cells. All operation procedures were strictly performed in accordance with the operation manual proposed by Army Medical University.

### Cytotoxicity assay

In this study, we used the WKYMVm peptide as an activator of the FPR2 pathway, so it was necessary to detect the toxicity of the WKYMVm peptide. We used CCK-8 to assess the toxicity of the WKYMVm peptide on mBMSCs. According to previous studies [22], we chose 1 μmol/L as the concentration of WKYMVm peptide and 10 μmol/L as the concentration of WRW4 peptide. mBMSCs were seeded in 96-well plates at a density of 5×10^3^ per well. After one night of culture, the mBMSCs were exposed to WKYMVm peptide and observed for 24, 48, and 72 h. Next, we added CCK-8 solution (100 μl/well) to 96-well plates and left them in the dark for another hour. Finally, we detected and recorded the absorbance at 450 nm with a microplate reader and compared it with the control group without WKYMVm peptide.

### Preparation of WKYMVm peptide-conditioned medium and WKYMVm peptide plus WRW4 peptide-conditioned medium

One day after mBMSCs were stimulated by WKYMVm peptide or WKYMVm peptide plus WRW4 peptide, the supernatant was collected by centrifugation at 3000 rpm for 10 min. The collected supernatant was used as conditioned medium for growing RAW 264.7 cells in the follow-up study. In this study, the collected supernatant was called WKYMVm peptide-conditioned medium or WKYMVm peptide plus WRW4 peptide-conditioned medium.

### Western blotting

To detect protein expression, western blotting was used. β-Actin served as an internal control. According to the experimental design, mBMSCs, exosomes, or M2 macrophages were dissolved in a cell lysate containing PMSF. The proteins were transferred to polyvinylidene fluoride (PVDF) membranes by electrophoresis. The PVDF membrane was placed in the sealing liquid and shaken at low speed for 1 h. Next, the sealed PVDF membrane was placed in the antibody box containing the primary antibody and incubated at 4 °C overnight. Then, the PVDF membrane was washed with Tris-buffered saline Tween (TBST) thrice. Then, the PVDF membrane was placed into an antibody box containing the secondary antibody and incubated for 2 h at room temperature. We also washed the PVDF membrane thrice with TBST. Finally, we used the ChemiDoc XRS Imaging System (Bio-Rad, CA, USA) to capture images of protein bands and analyzed the image using the ImageJ software.

### RNA isolation and qRT-PCR

To investigate gene expression, RNA isolation and qRT-PCR were used. Glyceraldehyde 3-phosphate dehydrogenase (GAPDH) served as an internal control. mBMSCs were lysed with TRIzol buffer. Complementary DNA was synthesized from 1 μg of total RNA using a reverse transcription kit, and gene expression was detected by qRT-PCR.

### Lysozyme activity assay kit

We used alysozyme activity assay kit to detect lysosomal activity of mBMSCs according to the manufacturer’s instructions.

### Exosome extraction

To collect exosomes secreted by mBMSCs, a exoEasy Maxi Kit (QIAGEN GmbH, Hilden, Germany) was used. The exosome isolation technique fulfills the criteria of Minimal Information for Studies of Extracellular Vesicles 2018 (MISEV 2018) [23, 24]. In the continuous centrifugation stage, the first centrifugation was performed at 300 g for 5 min to remove cells. The second centrifugation was performed at 1200 g for 10 min, and the third centrifugation was performed at 10,000 g for 30 min to remove cellular debris. The exosomes were obtained from the supernatant according to the instructions of the exosome extraction kit.

### Immunofluorescence

Immunofluorescence was used to count the number of polarized M2 macrophages. The cells were fixed with 4% paraformaldehyde for 20 min. Next, the cells were permeated with 0.3% Triton X-100 solution for 15 min. Then, cells were covered with 5% bovine serum albumin (BSA) at 37 °C for 30 min. After the BSA was removed, we added the primary antibody and antibody diluents at a ratio of 1:200 into the plates overnight at 4 °C. The next day, we removed the primary antibody at room temperature. Then, we added secondary antibody and antibody diluents at a ratio of 1:400 to the plates. We incubated the cells at 37 °C for 30 min. Then, we removed the secondary antibody. The cells were incubated with 2-(4-amidinophenyl)-6-indolecarbamidine dihydrochloride (DAPI) solution at room temperature for 5 min. Finally, we added fluorescent mounting media to the plates and observed the cells under a fluorescence microscope. The quantification of M2 macrophage polarization was performed using ImageJ.

### Transfection

To prevent the effect of miRNA-146 in exosomes secreted by mBMSCs, we used a miRNA-146 inhibitor for transfection. The WKYMVm peptide-conditioned medium was transfected with miRNA-146 inhibitor and transfection reagent according to the manufacturer’s instructions.

### ISG15 expression assay

mBMSCs were seeded in 6-well plates at a density of 1×10^6^ per well. After one night of culture, WKYMVm peptide and WKYMVm peptide plus WRW4 peptide were added to different plates. After 24 h, we used western blotting and qRT-PCR (forward primer: TTTCCTGGTGTCCGTGA, reverse primer: TCTGGGCAATCTGCTTCT) to detect ISG15 expression. ISG15 expression in the WKYMVm peptide group was compared with that in the control group without the WKYMVm peptide and the WKYMVm peptide plus WRW4 peptide group.

### TFEB expression assay

mBMSCs were seeded in 6-well plates at a density of 1×10^6^ per well, and WKYMVm peptide and WKYMVm peptide plus WRW4 peptide were added to different plates the next day. After 24 h, we used western blotting and qRT-PCR (forward primer: AAGAACAGGGGTGAGGCA, reverse primer: CCCAGGCTCAGGAGAGG) to detect TFEB expression. TFEB expression in the WKYMVm peptide group was compared with that in the control group without WKYMVm peptide and the WKYMVm peptide plus WRW4 peptide group.

### Lysosomal activity assay

mBMSCs were seeded in 96-well plates at a density of 5×10^3^ per well, and WKYMVm peptide and WKYMVm peptide plus WRW4 peptide were added to different plates the next day. After 24 h, lysosomal activity was detected. The lysosomal activity of the WKYMVm peptide group was compared with that of the control group without the WKYMVm peptide and the WKYMVm peptide plus WRW4 peptide group.

### Exosome content assay

Exosomes secreted by MSCs contain abundant miRNA-146, which could promote the polarization of M2 macrophages [19–21]. mBMSCs were seeded in 6-well plates at a density of 1×10^6^ per well. After one night of culture, WKYMVm peptide and WKYMVm peptide plus WRW4 peptide were added to different plates. After 24 h, we centrifuged the cell culture medium continuously. After that, we used an exosome extraction kit to collect exosomes. Then, the exosomes were observed under transmission electron microscopy (JEM-1400PLUS, Japan) and analyzed by western blotting (marker: CD9 and CD63). Finally, we compared the exosome content of the WKYMVm group with that of the control group without the WKYMVm peptide and the WKYMVm peptide plus WRW4 peptide group.

### Assay of M2 macrophage polarization promoted by exosomes secreted by mBMSCs

In addition, we counted the number of polarized M2 macrophages. We cultured RAW 264.7 cells in WKYMVm peptide-conditioned medium, medium without WKYMVm peptide and WKYMVm peptide plus WRW4 peptide-conditioned medium. After 24 hours, we used immunofluorescence (marker: CD206) and western blotting (marker: CD206, Arg-1) to count the number of polarized M2 macrophages. First, RAW 264.7 cells were seeded in 96-well plates at a density of 5×10^3^ per well for immunofluorescence and 6-well plates at a density of 1×10^6^ per well for western blotting. The next day, the WKYMVm peptide-conditioned medium, the medium without WKYMVm peptide and the WKYMVm peptide plus WRW4 peptide-conditioned medium were removed. Then, we washed the plates with PBS twice. After immunofluorescence and western blotting were completed, the number of polarized M2 macrophages in the WKYMVm peptide-conditioned medium group was compared with the medium without WKYMVm peptide group and the WKYMVm peptide plus WRW4 peptide-conditioned medium group.

### Analysis of M2 macrophage polarization after inhibiting miRNA-146 in exosomes secreted by mBMSCs

To investigate whether miRNA-146 in exosomes secreted by mBMSCs could promote M2 macrophage polarization, we cultured RAW 264.7 cells in WKYMVm peptide-conditioned medium and WKYMVm peptide-conditioned medium supplemented with miRNA-146 inhibitor. RAW 264.7 cells were seeded in 6-well plates at a density of 1×10^6^cells per well. After 24 h, we used western blotting (marker: CD206, Arg-1) to count the number of polarized M2 macrophages. Then, the number of polarized M2 macrophages in the WKYMVm peptide-conditioned medium group was compared with that in the WKYMVm peptide-conditioned medium supplemented with miRNA-146 inhibitor group.

### Statistical analysis

All the data were obtained from three independent researchers. All data are expressed as the mean ± standard deviation (SD). Student’s *t* test was used to compare the differences between the two groups. All statistical analyses were performed by the SPSS 22.0 software, and a *P* value less than 0.05 was considered statistically significant. In addition, all figures were generated using the GraphPad 8.4.0 software. (*P<0.05, **P<0.01, ***P<0.001, N.S indicates not significant)

## Results

### WKYMVm peptide mediated cytotoxicity in mBMSCs

**Fig. 1:**
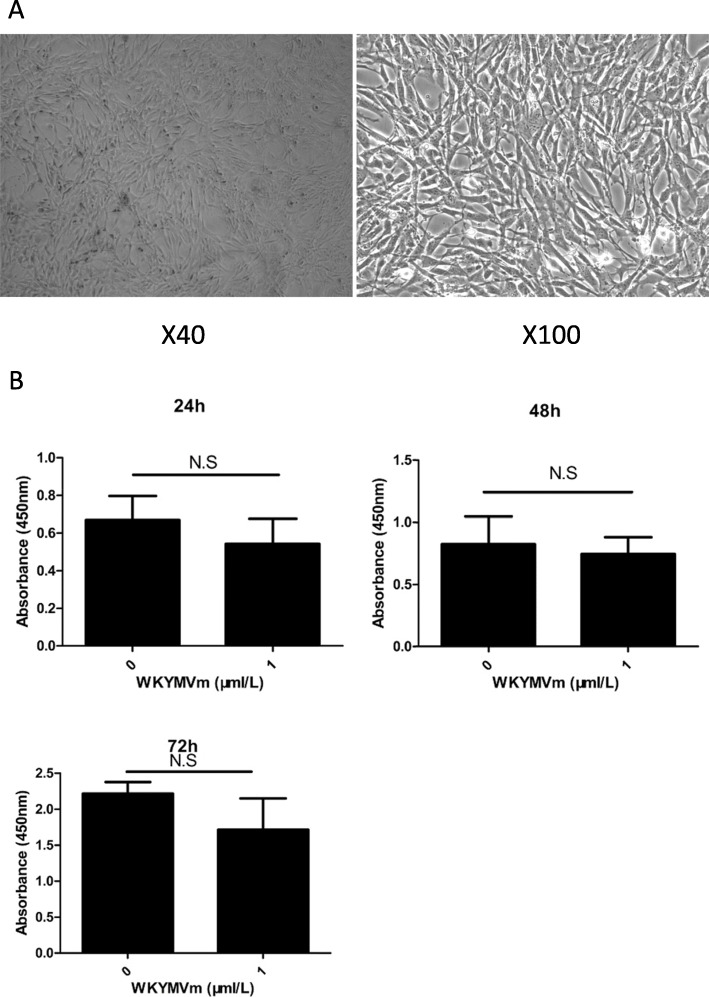
The cytotoxicity of WKYMVm peptide on mBMSC at 24, 48, and 72 h. **a** Morphology of mBMSCs under optical microscope. **b** The proliferation of mBMSCs, treated with WKYMVm peptide (1 μmol/L), was detected by CCK-8. Data represent means ± SD. N.S relative to controls in which the concentration of WKYMVm peptide was 0 μmol/L

mBMSCs (Fig. 1a) were incubated with WKYMVm peptide for 24, 48, and 72 h. Next, we used CCK-8 to detect whether mBMSC proliferation and viability were changed. We found that the WKYMVm peptide did not affect mBMSC proliferation and viability (Fig. 1b) compared with the control group without the WKYMVm peptide.

### WKYMVm peptide activated the FPR2 pathway in mBMSCs, resulting in decreased ISG15 expression

**Fig. 2:**
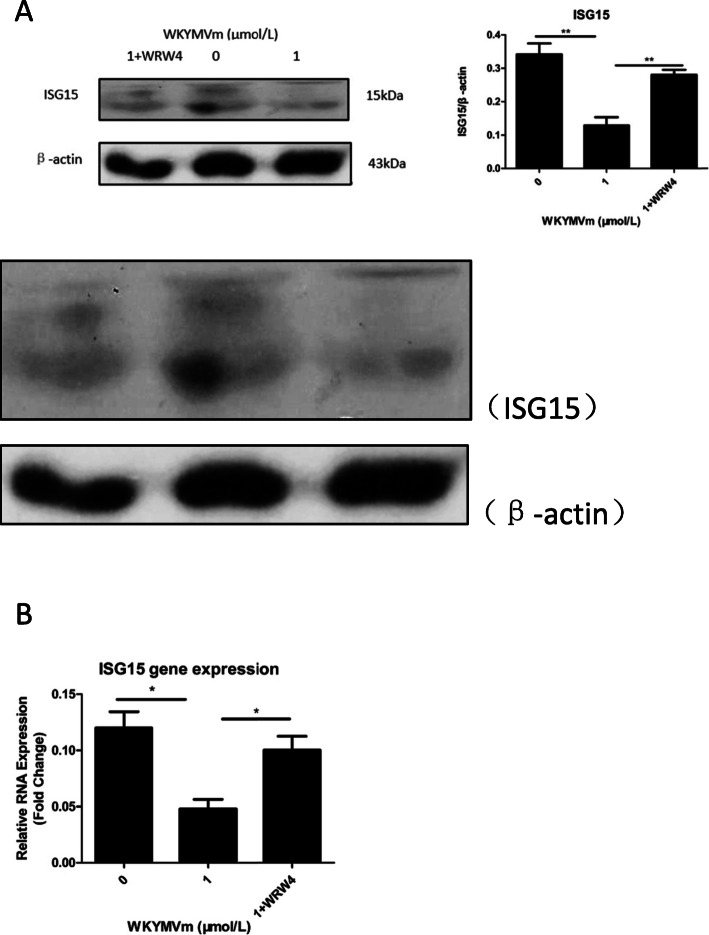
WKYMVm peptide reduced ISG15 expression through activating FPR2 pathway in mBMSC. mBMSCs were seeded in 6-well plates and cultured with WKYMVm peptide (1 μmol/L) or WKYMVm peptide (1 μmol/L) plus WRW4 peptide (10 μmol/L) for 24 h. **a** ISG15 protein level was analyzed using western blotting. **b** The relative RNA expression of ISG15 was determined by qRT-PCR. Data represent means ± SD. *P < .05, **P < .01, and ***P < .001 relative to controls in which the concentration of WKYMVm peptide was 0 μmol/L

According to our previous study, the WKYMVm peptide could activate the FPR2 pathway in MSCs [22]. In this study, we found that when the FPR2 pathway was activated, the expression of ISG15 in mBMSCs was decreased significantly compared with that in the control group without the WKYMVm peptide (Fig. 2a, b). WRW4 peptide, an inhibitor of the FPR2 pathway, blocked the decreasing effect of ISG15 expression induced by WKYMVm (Fig. 2a, b).

### Activation of the FPR2 pathway by the WKYMVm peptide decreased TFEB expression

**Fig. 3:**
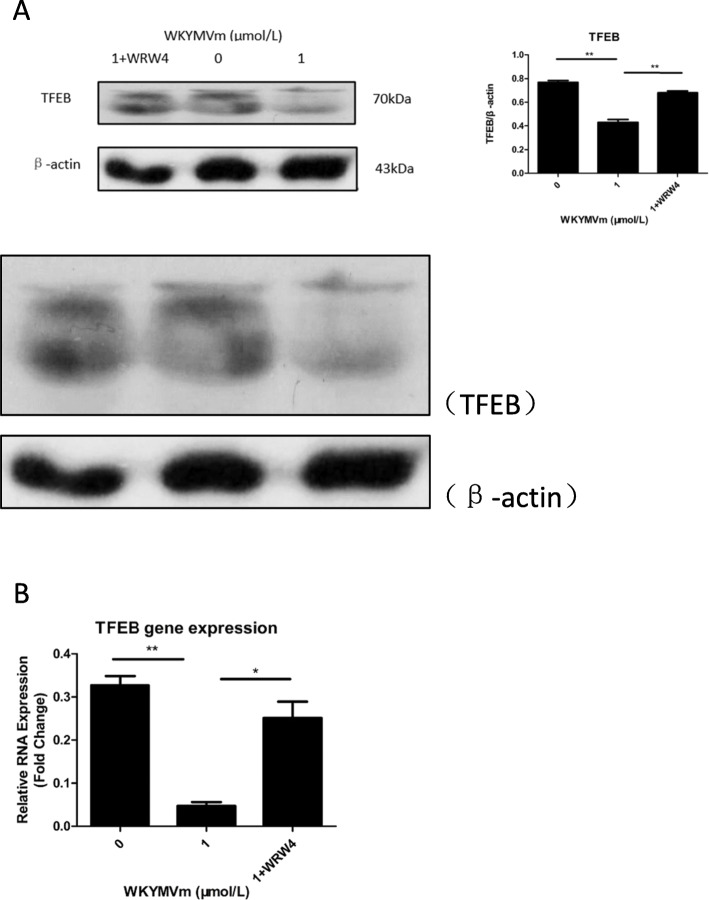
WKYMVm peptide downregulated the expression of TFEB in mBMSC by combination with FPR2 pathway. mBMSCs incubated with WKYMVm peptide (1 μmol/L) or WKYMVm peptide (1 μmol/L) plus WRW4 peptide (10 μmol/L) for 24 h. **a** Western blotting carried out with TFEB and β-actin special antibodies to determine the TFEB protein expression level. **b** qRT-PCR was used to analyze the relative RNA expression of TFEB. Data represent means ± SD. *P < .05, **P < .01, and ***P < .001 relative to controls in which the concentration of WKYMVm peptide was 0 μmol/L

TFEB, an intracellular transcription promoter, is also an important factor in activating lysosomal activity. By detecting TFEB expression in mBMSCs, we found that compared with the control group without WKYMVm peptide, TFEB expression was decreased in the WKYMVm peptide group (Fig. 3a, b). In addition, WRW4 peptide, an inhibitor of the FPR2 pathway, blocked the decreasing effect of TFEB expression induced by WKYMVm (Fig. 3a, b).

### Activation of the FPR2 pathway in mBMSCs by the WKYMVm peptide led to decreased lysosomal activity

**Fig. 4:**
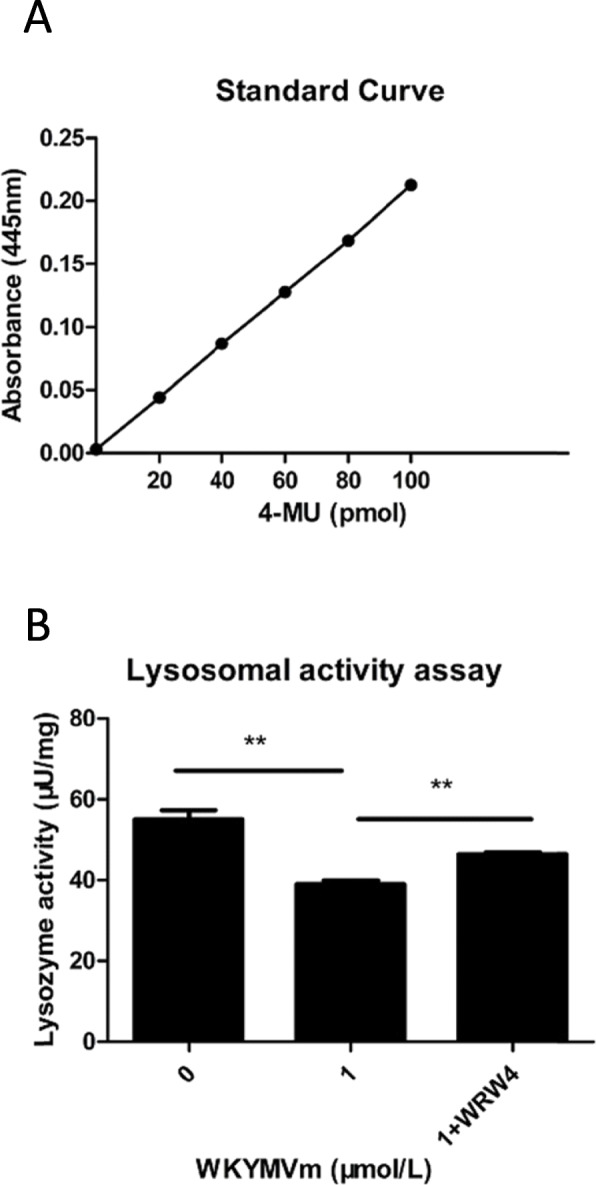
WKYMVm peptide decreased the lysosomal activity of mBMSC. mBMSCs incubated with WKYMVm peptide (1 μmol/L) or WKYMVm peptide (1 μmol/L) plus WRW4 peptide (10 μmol/L) for 24 h. **a** Standard curve drawn according to the manufacturer’s instructions. **b** Effects of WKYMVm peptide on lysosomal activity (lysosomal activity=(*B*/Δ*T*×*V*)×*D*. *B*=amount of 4-methylumbelliferone (4-MU) in sample well calculated using the standard curve (pmol); Δ*T*=reaction time (min), 30 min in our study; *V*=sample volume added into the reaction well (ml), 0.02 ml in our study; *D*=sample dilution factor, 1 in our study). Data represent means ± SD. *P < .05, **P < .01, and ***P < .001 relative to controls in which the concentration of WKYMVm peptide was 0 μmol/L

In our study, we found that FPR2 pathway activation in mBMSCs by the WKYMVm peptide led to a decrease in lysosomal activity compared with the control group without the WKYMVm peptide (Fig. 4a, b). In addition, the WRW4 peptide, an inhibitor of the FPR2 pathway, blocked the decrease in lysosomal activity induced by WKYMVm (Fig. 4a, b).

### Activation of the FPR2 pathway by the WKYMVm peptide promoted exosome secretion by mBMSCs

**Fig. 5:**
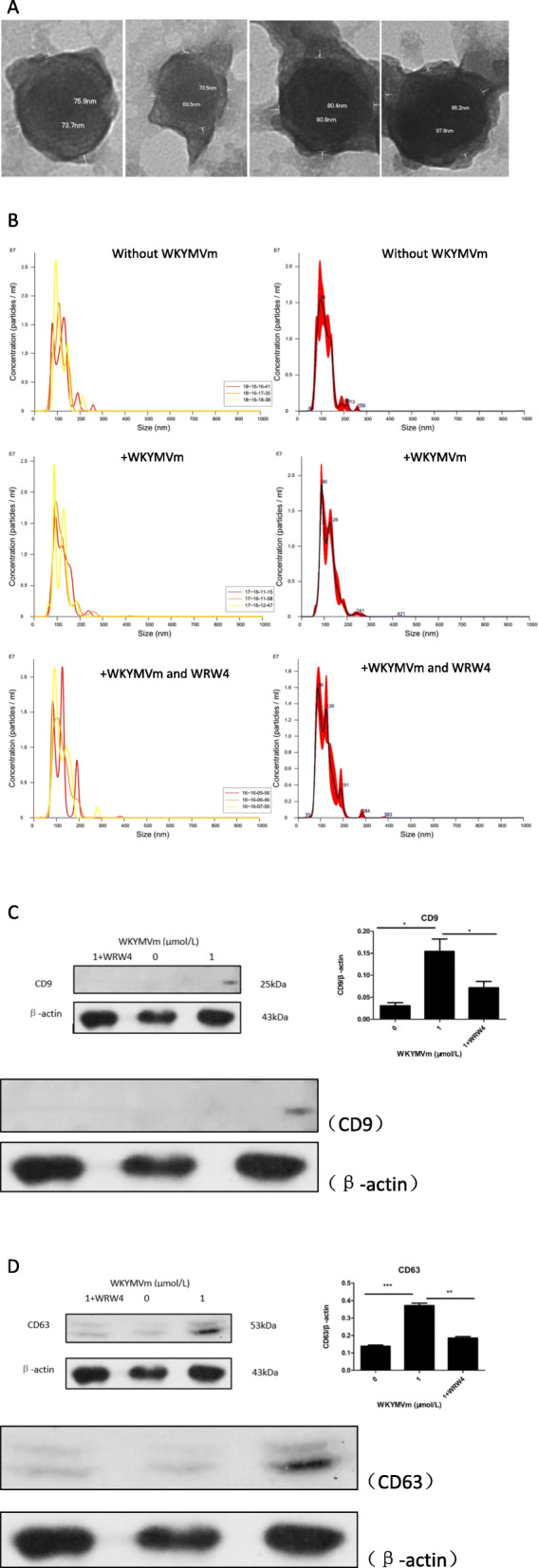
WKYMVm peptide increased the exosome secretion of mBMSC. mBMSCs incubated with WKYMVm peptide (1 μmol/L) or WKYMVm peptide (1 μmol/L) plus WRW4 peptide (10 μmol/L) for 24 h. **a** Morphology of exosomes secreted by mBMSCs under the transmission electron microscopy. **b** Size distribution and concentrations of the particles in isolated exosome fractions under NTA. **c** and **d** The expression levels of exosome makers CD9 and CD63 were detected by western blotting. Data represent means ± SD. *P < .05, **P < .01, and ***P < .001 relative to controls in which the concentration of WKYMVm peptide was 0 μmol/L

In this study, we detected the content of exosomes secreted by mBMSCs and compared them with the control group without WKYMVm peptide. The morphology is shown in Fig. 5a. The size distribution and concentrations of the particles in isolated exosome fractions is measured by nanoparticle tracking analysis (NTA) and shown in Fig. 5b. We found that the content of exosomes secreted by mBMSCs was increased with the WKYMVm peptide (Fig. 5c, d). In addition, the WRW4 peptide, an inhibitor of the FPR2 pathway, blocked the increasing effect of exosome secretion induced by WKYMVm (Fig. 5c, d).

### Exosomes secreted by mBMSCs promote the polarization of M2 macrophages

**Fig. 6:**
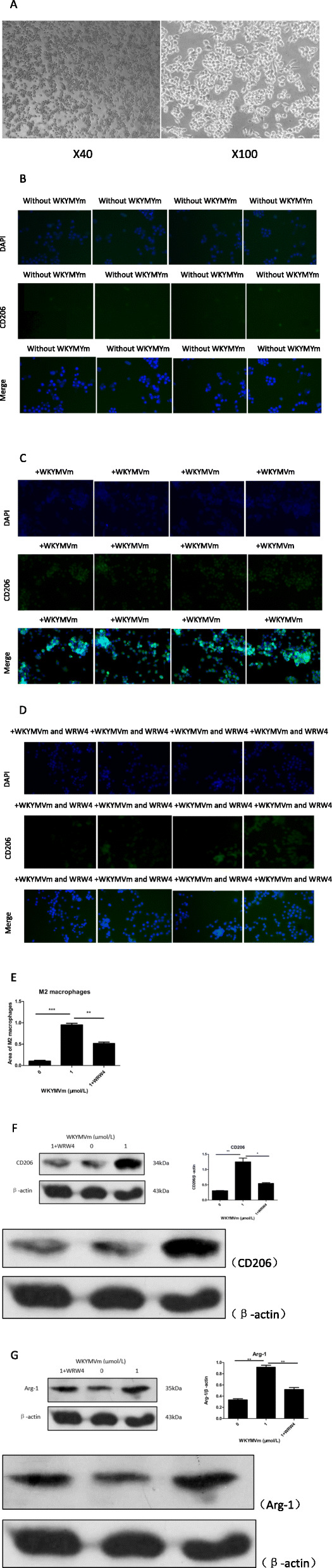
The exosomes secreted by mBMSC accelerated M2 macrophage polarization. RAW 264.7 cells were cultured with exosomes secreted by mBMSCs which incubated with WKYMVm peptide (1 μmol/L) or WKYMVm peptide (1 μmol/L) plus WRW4 peptide (10 μmol/L) for 24 h. (**a**) Morphology of RAW 264.7 cells under optical microscope. DAPI (blue), CD206 (green), and merge (blue/green) were observed by immunofluorescence microscopy in (**b**) (control group), (**c**) (WKYMVm peptide group), and (**d**) (WKYMVm peptide plus WRW4 peptide group). (**e**) The quantification of M2 macrophage polarization was observed by immunofluorescence (Marker: CD206). (**f** and **g**) The quantification of CD206 and Arg-1 protein expression levels which was the makers of M2 macrophage was analyzed by western blotting. Data represent means ± SD. *P < .05, **P < .01, and ***P < .001 relative to controls in which the concentration of WKYMVm peptide was 0 μmol/L

We cultured RAW 264.7 cells (Fig. 6a) in WKYMVm peptide-conditioned medium, medium without WKYMVm peptide, and WKYMVm peptide plus WRW4 peptide-conditioned medium. We found that the WKYMVm peptide-conditioned medium promoted M2 macrophage polarization compared with the medium without WKYMVm peptide (Fig. 6b, c, d, e, f, g). In addition, WRW4 peptide, an inhibitor of the FPR2 pathway, blocked the increasing effect of M2 macrophage polarization induced by WKYMVm peptide-conditioned medium (Fig. 6b, c, d, e, f, g).

### miRNA-146 in exosomes secreted by mBMSCs promoted M2 macrophage polarization

**Fig. 7:**
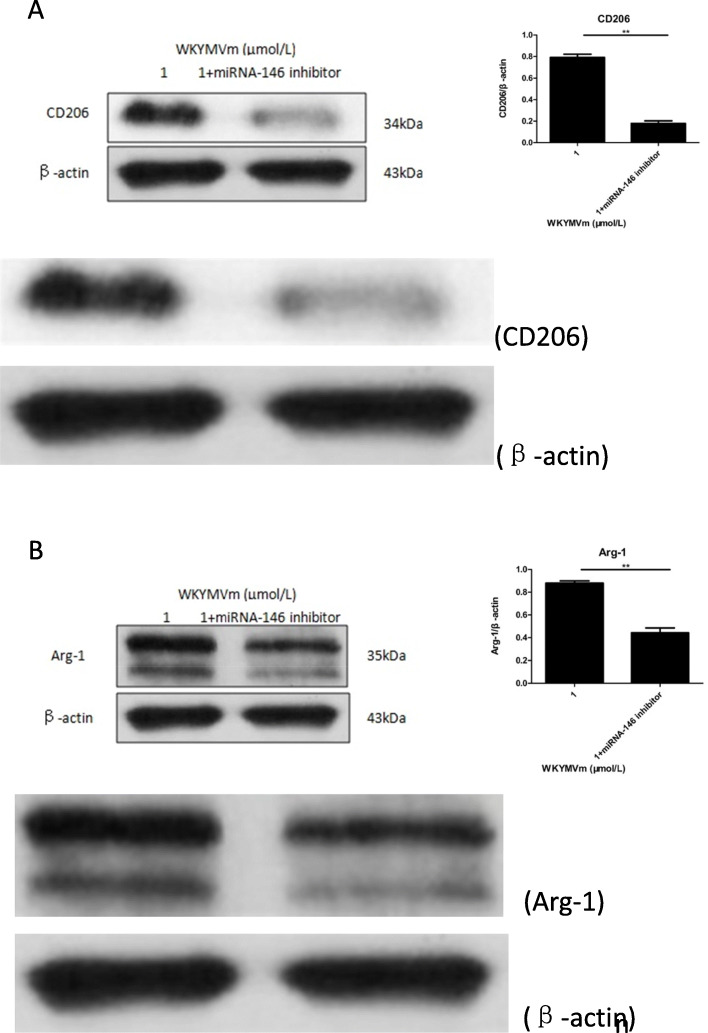
The miRNA-146 in exosomes secreted by mBMSC could promote M2 macrophage polarization. RAW 264.7 cells were cultured with exosomes secreted by mBMSCs which incubated with WKYMVm peptide (1 μmol/L) or WKYMVm peptide (1 μmol/L) supplemented with miRNA-146 inhibitor for 24 h. **a** and **b** The quantification of M2 macrophage markers (CD206 and Arg-1) was measured by western blotting. Data represent means ± SD

We cultured RAW 264.7 cells (Fig. 6a) in WKYMVm peptide-conditioned medium and WKYMVm peptide-conditioned medium supplemented with miRNA-146 inhibitor. We found that WKYMVm peptide-conditioned medium supplemented with miRNA-146 inhibitor blocked the polarization of M2 macrophages compared with WKYMVm peptide-conditioned medium (Fig. 7a, b).

## Discussion

Exosomes, originally discovered in the 1980s, are vesicles secreted by cells with a diameter of approximately 30-100 nm that participate in a variety of cell activities [25]. Bone-derived exosomes can be secreted by a variety of cells, including osteocytes, osteoblasts, osteoclasts, and MSCs [26]. Many studies have demonstrated that bone-derived exosomes regulate the behavior of other cells by regulating cell communication and transporting cellular active substances [27]. Furthermore, exosomes contain many biological molecules, such as proteins, enzymes, and miRNAs, which have important physiological or pathological effects [28]. On the other hand, according to our previous studies, the WKYMVm peptide, as a powerful activator of the FPR2 pathway, activates the FPR2 pathway in MSCs [22]. However, when the FPR2 pathway of MSCs is activated, the changes that occur, the impact on the secreted exosomes of MSCs, and the effects of secreted exosomes on macrophage differentiation are rarely reported. According to the current research results, we demonstrate that M2 macrophages promote local vascularization, and local vascularization provides a solution for repairing bone defects [8, 9]. Therefore, in this study, we mainly found that when the FPR2 pathway of mBMSCs was activated by the WKYMVm peptide, a series of changes promoted the secretion of exosomes, and the exosomes secreted by mBMSCs could ultimately promote M2 macrophage polarization.

At present, many scholars have noted that M2 macrophages promote local vascularization, and the increase in local vascularization is the basis of bone defect repair [8, 9]. Our studies found that mBMSCs increase exosome secretion through a series of reactions by activating the FPR2 pathway, and that the exosomes were rich in miRNA-146, which was the key to promoting the polarization of M2 macrophages [19–21]. First, the WKYMVm peptide activates the FPR2 pathway in mBMSCs and decreases the expression of the inflammatory-related ubiquitin-like protein ISG15. We confirmed the above hypothesis by measuring the expression of ISG15 in mBMSCs and comparing it with the control group without the WKYMVm peptide. Moreover, the decreased expression of ISG15 induced by the activation of the FPR2 pathway by the WKYMVm peptide was blocked by the WRW4 peptide. Second, activation of the FPR2 pathway can lead to reduced TFEB expression in mBMSCs and the release of more exosomes. Similarly, we confirmed the above hypothesis by measuring the expression of TFEB and comparing it with the control group without the WKYMVm peptide. We also found that the decreased expression of TFEB induced by the activation of the FPR2 pathway by the WKYMVm peptide was blocked by the WRW4 peptide. Through further research, we confirmed that by decreasing the expression of ISG15 and TFEB, lysosomal activity was also decreased. Unsurprisingly, the decrease in lysosomal activity upon activation of the FPR2 pathway by the WKYMVm peptide was blocked by the WRW4 peptide.

There are three stages of exosome secretion. Stage one: The inner membrane of endosomes forms luminal vesicles. Stage two: When the content of luminal vesicles reaches a certain level, the vesicles begin to gather together and fuse into multicystic vesicles. Stage three: Multicystic vesicles, also known as precursors of exosomes, either fuse with lysosomes and decompose or fuse with the plasma membrane and are released from the cell [29, 30]. In our study, increased exosome secretion mainly occurred by regulating the third stage, i.e., changing lysosomal activity. We compared the content of exosomes secreted by mBMSCs in the WKYMVm peptide group with that in the control group without the WKYMVm peptide and confirmed that FPR2 pathway activation promotes the secretion of exosomes by mBMSCs. In addition, the increasing content of exosomes secreted by mBMSCs induced by the activation of the FPR2 pathway by the WKYMVm peptide was blocked by the WRW4 peptide. Finally, we investigated the effect of exosomes secreted by mBMSCs on the polarization of M2 macrophages. According to the previous theory, exosomes secreted by MSCs contain abundant miRNA-146, which can promote the polarization of M2 macrophages [19–21]. In our study, we cultured RAW 264.7 cells in WKYMVm peptide-conditioned medium, medium without WKYMVm peptide, and WKYMVm peptide plus WRW4 peptide-conditioned medium. Then, we counted the number of polarized M2 macrophages in the above three groups. We found that the secreted exosomes of mBMSCs promoted the polarization of M2 macrophages. In addition, the increased number of polarized M2 macrophages induced by WKYMVm peptide-conditioned medium could also be inhibited by the WRW4 peptide. Furthermore, we cultured RAW 264.7 cells in WKYMVm peptide-conditioned medium and WKYMVm peptide-conditioned medium supplemented with miRNA-146 inhibitor. Through the comparison between the above two groups, we confirmed the effect of miRNA-146 in the exosomes secreted by mBMSCs on promoting the polarization of M2 macrophages.

Promoting local vascularization is the premise of the treatment of bone defects, and M2 macrophages have a clear role in promoting local vascularization [31]. It has been reported that even if MSCs are removed, the polarization of M2 macrophages can continue for a period of time [32, 33], indicating that the exosomes secreted by MSCs play a very important role in the polarization of M2 macrophages.

**Fig. 8:**
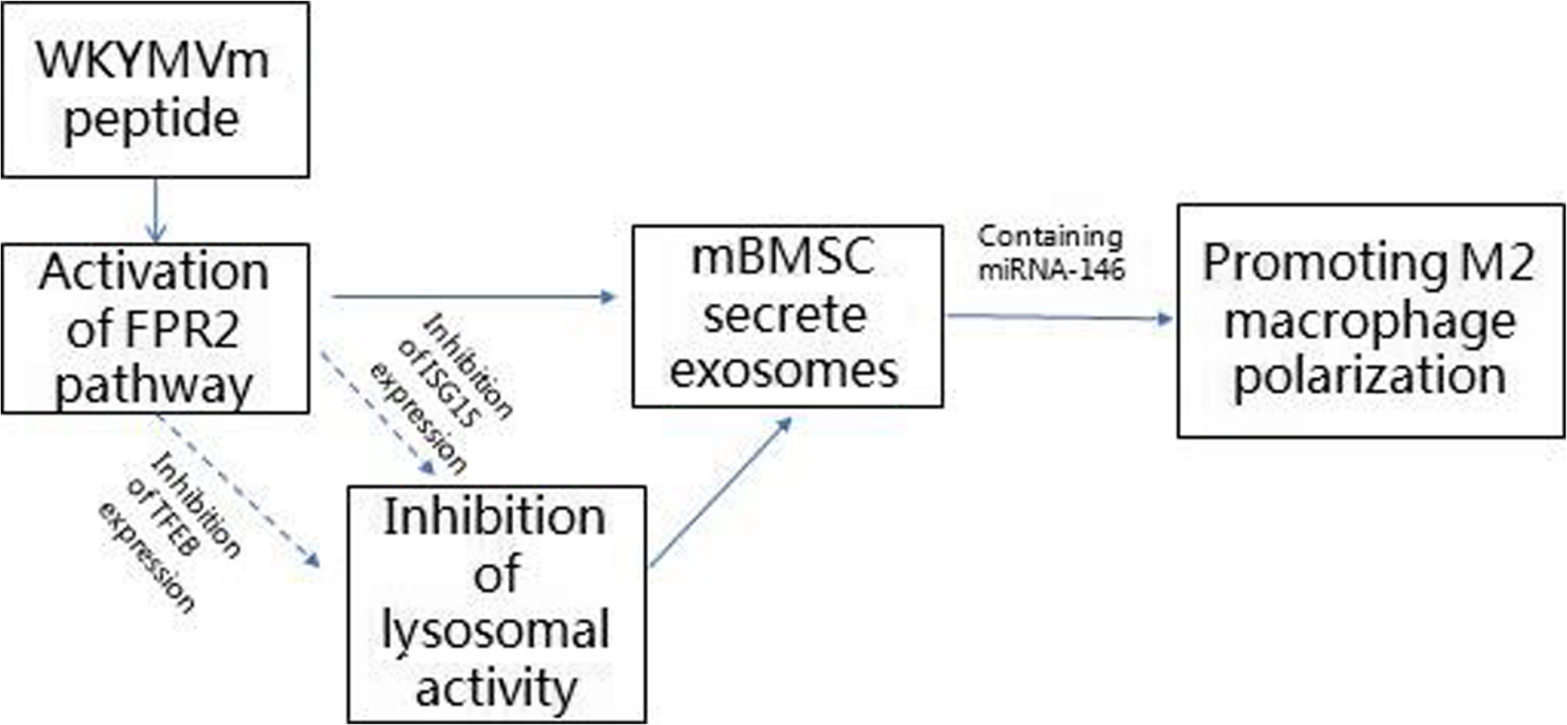
Schematic diagram of exosome secretion by mBMSCs and M2 macrophage polarization stimulated by exosomes (solid line represents activation; dotted line represents inhibition)

In the inflammatory environment, the expression of many inflammatory-related proteins increases [34, 35]. For example, lipoproteins (LPS) are involved in a variety of inflammatory reactions [36, 37]. We found that when the FPR2 pathway of mBMSCs was activated, the effect was opposite of that of the LPS-induced response. In the LPS-induced inflammatory response, IL-1β, tumor necrosis factor-α (TNF-α), IL-6, and other classic inflammatory factors are released in large quantities, and these proinflammatory factors activate multiple inflammation-related pathways, including the nuclear transcription factor-κB (NF-κB), mitogen-activated protein kinase (MAPK), and signal transducer and activator of transcription (STAT) signaling pathways [35–37]. This results in increased ubiquitin-like protein ISG15 expression and subsequently affects the secretion of exosomes by MSCs [38, 39]. Interestingly, the STAT3 pathway, a subtype of the STAT pathway, plays an important role in the LPS-induced inflammatory response [40, 41]. According to previous studies, IL-6 combines with glycoprotein 130 (gp130) and transforms into membrane-bound IL-6 receptor (Mil-6R), which activates the STAT3 pathway [42]. Further studies demonstrate that CD9 prevents members of the STAT3 pathway from being degraded by ubiquitin-dependent lysosomes and stabilizes gp130 [43]. Therefore, we believe that in an inflammatory environment, when CD9 expression is blocked, the IL-6/gp130/STAT3 pathway is affected, eventually leading to a decrease in ISG15 expression. Therefore, we suggest that the WKYMVm peptide activates the FPR2 pathway in mBMSCs and inhibits the binding of IL-6 with gp130 to reduce the expression of CD9, achieving the positive regulation of exosome secretion. However, the activation of the FPR2 pathway in MSCs has an anti-inflammatory effect, which resists the LPS-induced inflammatory response, thus resulting in decreased ubiquitin-like protein ISG15 expression and positively regulating exosome secretion by MSCs [44–46]. The mechanism of exosome secretion by mBMSCs and M2 macrophage polarization stimulated by exosomes is shown in Fig. 8.

## Conclusions

In conclusion, our study demonstrated that after activation of the FPR2 pathway in mBMSCs, the secretion of exosomes was promoted by regulating ISG15 and TFEB expression. Decreased ISG15 and TFEB expression led to decreased lysosomal activity.

In addition, our study also confirmed that exosomes secreted by mBMSCs promote the polarization of M2 macrophages and the function of miRNA-146 in exosomes secreted by mBMSCs. Because M2 polarization promotes local vascularization [47, 48], our study provides a new idea for the treatment of bone defects. The regulation of secretion of MSC exosomes is a future research direction.

However, our study still has some limitations. First, our studies focused on MSCs of mouse bone marrow. Although it has been confirmed that the activation of the FPR2 pathway promotes the secretion of exosomes and eventually lead to polarization of M2 macrophages, further research is needed to demonstrate whether human MSCs are also regulated by this mechanism. Second, the mechanism of local vascularization after M2 macrophage polarization still requires further study. Finally, it is necessary to establish relevant animal models to clarify the detailed mechanism by which local vascularization promotes bone defect repair through animal experiments.

## Data Availability

The data used to support the findings of this study are available from the corresponding author upon request.

## Abbreviations

MSCs: Mesenchymal stem cells
mBMSC: Murine bone marrow-derived MSCs
FPR: Formyl peptide receptors
CCK-8: Cell Counting Kit-8
WB: Western blotting
qRT-PCR: Quantitative real-time polymerase chain reaction
ISG15: Interferon stimulated gene 15
TFEB: Transcription factor EB
Th2: T helper 2 cell
IL: Interleukin
TGF: Transforming growth factor
ATCC: American Type Culture Collection
DMEM: Dulbecco’s Modified Eagle’s Medium
FBS: Fetal bovine serum
PBS: Phosphate buffered saline
DMSO: Dimethyl sulfoxide
RIPA: Radio immunoprecipitation assay
PMSF: Phenylmethanesulfonyl fluoride
PVDF: Polyvinylidene fluoride
TBST: Tris-buffered saline Tween
GAPDH: Glyceraldehyde 3-phosphate dehydrogenase
MISEV: Minimal information for studies of extracellular vesicles
BSA: Bovine serum albumin
DAPI: 2-(4-Amidinophenyl)-6-indolecarbamidine dihydrochloride
SD: Standard deviation
N.S: Not significant
NTA: Nanoparticle tracking analysis
4-MU: 4-Methylumbelliferone
TNF: Tumor necrosis factor
LPS: Lipoproteins
NF-κB: Nuclear transcription factor κB
MAPK: Mitogen-activated protein kinase
STAT: Signal transducer and activator of transcription
gp130: Glycoprotein 130
Mil-6R: Membrane-bound IL-6 receptor
